# iMHere: A Novel mHealth System for Supporting Self-Care in Management of Complex and Chronic Conditions

**DOI:** 10.2196/mhealth.2391

**Published:** 2013-07-11

**Authors:** Bambang Parmanto, Gede Pramana, Daihua Xie Yu, Andrea D Fairman, Brad E Dicianno, Michael P McCue

**Affiliations:** ^1^Department of Health Information ManagementSchool of Health & Rehabilitation SciencesUniversity of PittsburghPittsburgh, PAUnited States; ^2^Department of Rehabilitation Science & TechnologySchool of Health & Rehabilitation SciencesUniversity of PittsburghPittsburgh, PAUnited States; ^3^Department of Physical Medicine & RehabilitationSchool of MedicineUniversity of PittsburghPittsburgh, PAUnited States

**Keywords:** mobile health, mhealth, self-care, clinician-directed self-care, self-management, telehealth, telemedicine, smartphone, chronic disease management, spina bifida, patient-clinician communications

## Abstract

**Background:**

Individuals with chronic conditions are vulnerable to secondary complications that can be prevented with adherence to self-care routines. They benefit most from receiving effective treatments beyond acute care, usually in the form of regular follow-up and self-care support in their living environments. One such population is individuals with spina bifida (SB), the most common permanently disabling birth defect in the United States. A Wellness Program at the University of Pittsburgh in which wellness coordinators supervise the care of individuals with chronic disease has produced remarkably improved outcomes. However, time constraints and travel costs have limited its scale. Mobile telehealth service delivery is a potential solution for improving access to care for a larger population.

**Objective:**

The project’s goal was to develop and implement a novel mHealth system to support complex self-care tasks, continuous adherence to regimens, monitoring of adherence, and secure two-way communications between patients and clinicians.

**Methods:**

We developed and implemented a novel architecture of mHealth system called iMHere (iMobile Health and Rehabilitation) consisting of smartphone apps, a clinician portal, and a two-way communication protocol connecting the two. The process of implementing iMHere consisted of: (1) requirement analysis to identify clinically important functions that need to be supported, (2) design and development of the apps and the clinician portal, (3) development of efficient real-time bi-directional data exchange between the apps and the clinician portal, (4) usability studies on patients, and (5) implementation of the mHealth system in a clinical service delivery.

**Results:**

There were 9 app features identified as relevant, and 5 apps were considered priority. There were 5 app features designed and developed to address the following issues: medication, skin care, bladder self-catheterization, bowel management, and mental health. The apps were designed to support a patient’s self-care tasks, send adherence data to the clinician portal, and receive personalized regimens from the portal. The Web-based portal was designed for clinicians to monitor patients’ conditions and to support self-care regimens. The two-way communication protocol was developed to facilitate secure and efficient data exchange between the apps and the portal. The 3 phases of usability study discovered usability issues in the areas of self-care workflow, navigation and interface, and communications between the apps and the portal. The system was used by 14 patients in the first 6 months of the clinical implementation, with 1 drop out due to having a poor wireless connection. The apps have been highly utilized consistently by patients, even those addressing complex issues such as medication and skincare. The patterns of utilization showed an increase in use in the first month, followed by a plateau.

**Conclusions:**

The system was capable of supporting self-care and adherence to regimen, monitoring adherence, supporting clinician engagement with patients, and has been highly utilized.

## Introduction

Individuals with chronic conditions account for 75% of health care expenditures in the United States [1-3]. Globally, chronic conditions currently account for 60% of the global disease burden, and this figure is expected to reach 80% by 2020 [4]. The global shortage of health care workers coupled with increasing life expectancy have made it a high priority of health care systems worldwide to develop innovative strategies to improve care for chronic conditions and to prevent secondary complications [4]. Innovative approaches to chronic care have been seen as key to improving health care and reducing cost. Individuals with chronic conditions are vulnerable to such secondary complications as infections, amputations, wounds, and depression. A recent prospective study has identified those secondary complications as the strongest predictors of risk for premature death for people with chronic conditions [5]. Evidence from around the world suggests that people with chronic conditions benefit most when they receive effective treatments beyond acute care, usually in the form of regular follow-up and self-management support in their living environments. Patients with effective self-management skills make better use of health care services, have improved health behaviors, and health status [6].

Individuals with spina bifida (SB) make up one of the chronic condition populations that is susceptible to hospitalization due to problems related to sepsis from urinary tract infections (UTIs) and skin wounds. People with SB are also susceptible to high readmission rates within 30 days of discharge [7,8]. These secondary complications may be partially preventable with appropriate support and interventions to improve self-care skills [7]. Proactively, the Spina Bifida Association of Western Pennsylvania (SBAWP) initiated an in-person Wellness Pilot Program in which 2 wellness coordinators (WC), both registered nurses, supervised the care of 35 individuals with complex medical needs [9]. The WC identified the issues that a given patient faced and then designed an individualized plan of care by actively engaging the patient in the process. They helped to ensure that patients scheduled medical appointments, taught patients how to fill their own prescriptions, encouraged self-examinations for problems such as skin breakdowns, communicated health care provider recommendations to patients with cognitive impairments, activated needed community resources, and “checked in” on patients with home visits in order to identify new problems early. The WC role as the liaison and the director of care empowered the individuals to be responsible for their own care [9].

The program produced remarkably improved outcomes with respect to medical complications and health care utilization measures. Individuals in the SBAWP Wellness Program had shorter lengths of hospital stay, with admission rates of only 12.9% compared to the national rate of 26.9 % [10]. SBWAP Wellness participants also had lower rates of skin breakdown (9.7%) and UTIs (16.1%) compared to those in the general SBAWP population (35.5% and 35.2% respectively). As the cost of treating 1 Stage IV wound for community dwelling adults can exceed $124,000 per wound [11], this program’s reduction in the incidence of wounds alone would allow for a significant overall decrease in health care costs. Unfortunately, travel and time constraints prevented the program from including larger groups of patients or those in remote locations [9].

A mobile health (mHealth) system is one way to reduce these constraints. mHealth would allow WC to serve a larger number of patients and would make the Wellness Program not only cost-effective but also scalable. It would also improve access to health care by allowing WC to reach underserved patients, who may benefit most. Mobile phones are the most commonly carried devices for people with disabilities [12]. Portability of mobile phones into almost any environment makes them an ideal tool for self-management, monitoring, and two-way engagement with the clinician.

Although currently there is no mobile or Web framework for the management of SB, the issues related to managing SB are similar to those of other chronic conditions. The current approaches to mHealth in managing chronic conditions fall into 5 categories:

1. *Stand-alone local apps* [13,14]. This first type includes self-monitoring apps such as diet or nutrition tracking apps that are used by consumers but do not connect the user with a clinician [15].
2. *Monitoring and recording apps using store-and-forward data transfer* [16,17]. The second type includes mobile remote health monitoring, such as blood pressure or heart monitor [18], or other store-forward monitoring apps [19].
3. *Consumer referential medical apps.* The third type includes educational apps that aim to help consumers manage chronic conditions through early self-identification and management of symptoms. This type may include access to Web-based disease management systems from a smartphone [20].
4. *Text messaging for engaging patients* [21-26]. The forth type is the most widely used method to engage patient, using both the original type of cellphone as well as a smartphone [27].
5. *Simple voice call, either in person or using interactive voice response (IVR)* [28]. The fifth type is a more traditional way of engaging patients by calling their cellphones.

The interactive Mobile Health and Rehabilitation (iMHere) system is an innovative advancement to the existing systems on the market and will be a significant contribution to current research literature on mHealth. It will support self-care that is initiated by patients or is directed by clinicians. Chronic condition management requires self-care, frequent communications between patients and clinicians, and continuous adherence to and adjustment of complex treatment regimens [29]. Certain conditions such as wounds and infections can worsen in a matter of hours and the ability to understand potential causes often requires a two-way real time dialog and image exchanges between the patient and clinician. These requirements cannot be supported by current mHealth architecture. The seamless two-way communication offered by the iMHere system will allow the clinician to provide a personalized treatment model, address major weaknesses of already existing apps, and mHealth systems for chronic conditions [30].

## Methods

### Overview

The project objective was to develop a novel mHealth system to support self-management and personalized care delivery for individuals with chronic conditions. The iMHere system consists of smartphone apps, a Web-based clinician portal, and a two-way communication connecting the patients and clinicians. The apps were designed to empower patients to perform preventive self-care activities and can be tailored to each patient’s needs and daily routine. Instead of existing as isolated local apps, the apps were designed to send monitoring data to the portal and also receive self-care regimens being pushed from the portal. In this study, using a Web-based portal, the clinician (typically a nurse coordinator, social worker, case manager, or patient advocate) could monitor patients’ compliance with regimens and send self-care regimens that would then be delivered to the patient via the apps. This allowed the clinician to monitor a patient’s status and intervene as needed. Clinicians could use the portal to tailor a regimen or treatment plan for each and every patient (eg, scheduled medication, wound care instructions, etc) and the portal would push the plan to the smartphone apps in real time, an advancement over existing health portals, which cannot push data to the apps. Real-time interactive medical monitoring of patient self-care offers a powerful unique solution for patients living with chronic conditions, where cognitive as well as physical disabilities present significant barriers to effective self-care. This innovative and unique two-way data exchange protocol was also designed to work in rural or low-resource areas with a spotty data connection. Moreover, the system supports an innovative security mechanism which will erase data in the event of the phone being lost and meets all Health Insurance Portability and Accountability Act (HIPAA) regulations.

### Architecture

**Figure 1:**
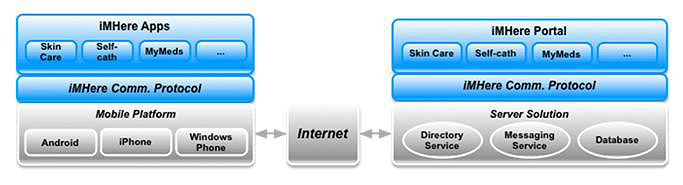
iMHere Architecture.

The architecture of iMHere consisted of smartphone apps on one side, a clinician portal on the other, and a two-way communication connecting the two, as illustrated in Figure 1. The upper layer of the architecture consists of the apps on the smartphone side and the portal application on the server side. Each app on the smartphone side had a corresponding application on the server portal side. For example, the MyMeds medication app had a corresponding application on the server side. The middle layer of the architecture consisted of the iMHere two-way communication protocol, which allowed real-time data exchange between the patient apps and clinician portal. The lower level of the architecture consisted of the operating systems on the smartphone and server, as well as the Internet protocol connecting the smartphone to the server. The architecture was designed to work with 3 major smartphone operating systems: Android, iOS, and Windows 8. However, since the apps needed to be rewritten for each OS, we implemented it only on an Android platform due to its open architecture, comparatively lower cost, and popularity. The apps and the clinician portal were developed using Java. Since the apps need to be available continuously for support, even if there is no Internet connection, the regimen data were also stored locally in the smartphone’s database. The communication protocol connected patients’ apps with the clinician portal and was used by the apps to send data to the portal and vice versa.

### Smartphone Apps

The purposes of the smartphone apps were three-fold: to support patients’ self-care tasks; to send adherence data to the clinician portal; to receive personalized regimens and education from the portal. In a series of meetings among the members of our team, including a physician, an occupational therapist, and software engineers, we identified important smartphone apps for patients based on our experience with the Wellness Program. There were 9 apps identified: medication management, skin care, appointments and scheduling, bowel management, bladder self-catheterization, exercise, mental health, nutrition and medical supplies. Of these 9, we selected the 5 apps that were considered crucial to decrease morbidity and mortality in the SB population apps: medication management, skin care, bowel management, bladder self-catheterization, and mental health.

#### Medication App

MyMeds helped patients follow their prescribed medication regimens by providing a reminder for every medication the patients were taking. Patients with SB are frequently prescribed several medications for the management of urinary incontinence, seizures, bowel management, depression, et cetera. Taking medications 3 or 4 times a day and consistently following the prescribed regimen is always challenging. This app helped patients keep track of all the medications they were currently taking or had taken in the past.

#### Skin Care App

Skin Care enabled patients to keep track of their skin problems and to communicate with clinicians on how to care for the skin problems. Loss of sensation in the lower body associated with the lesion of the spinal cord means there is no trigger that indicated a need to reposition oneself and to reduce the pressure on a particular part of the body. Therefore, people with SB had to be constantly vigilant for skin injury and breakdown over the lower body resulting from pressure ulcers. Poor circulation below the waist and improper functioning of the lymphatic system also cause the lower extremities to receive an inadequate supply of nutrients and oxygen, and to have a buildup of fluid. These combined issues mean that pressure ulcers can develop very quickly in people with SB, but they tend to heal very slowly [31]. Using the Skin Care app, patients could continuously monitor the progress of their skin conditions and report new wounds.

#### Mental Health App

Mood App allowed the patients to let the clinician know what type of mood related symptoms they were exhibiting and allowed the clinician to provide timely intervention for mental health problems. Studies have shown that, in comparison to the general population, people with SB are at a higher risk of depressed mood and lower self-worth, and they are more likely to think about suicide [32]. In a recent cross-cultural study [33], the incidence of depression was above 40%, while the incidence of anxiety was around 20%.

#### TeleCath

TeleCath is an app for bladder self catheterization management. This app reminded patients when it was time to perform bladder self-catheterization and to report potential problems encountered. Most people with SB have a neurogenic bladder. This means they are unable to perceive the sensation of bladder fullness, and they lack the neurologic integrity to have coordinated contraction of the bladder muscle and opening of the bladder sphincter [34]. Many people with SB have uninhibited bladder contractions, which may be accompanied by high bladder pressure. Some people may be able to empty their bladders partially by straining, but the emptying is incomplete. Even small amounts of residual urine in the bladder can lead to urinary tract infections. The combination of high bladder pressure and infection can place the kidneys at risk. A technique called Clean Intermittent Catheterization (CIC) prevents these problems by emptying the bladder several times a day via a tube inserted through the sphincter and into the bladder. CIC should occur 5 to 6 times during waking hours, which is every 3 to 4 hours throughout the day. Problems encountered during CIC are indicators of medical issues such as infection or injury. These are symptoms that can be reported via use of the app, including odor, pain, discoloration, or blood in the urine. UTIs and kidney infections require prompt medical attention, and the person will frequently need to follow up with antibiotics.

#### Bowel Management App

The Bowel Management App (BMQ) is an app for bowel management. The BMQ app helped to remind patients to perform their bowel program and report problems encountered. Bowel continence is important for maintaining skin integrity and is related to mental health issues (self-esteem), and social issues (quality of life and social isolation). Constipation can be a serious health concern as well. People with high spinal lesions have low internal sphincter pressure and rarely experience rectal sensation, while people with low spinal lesions have increased internal sphincter pressure and experience some rectal sensation [35]. Constipation is a result of having a neurogenic bowel and can also be attributed to inadequate intake of fluids, limited physical activity, lack of fiber in the diet, or as a side effect of a medication. Constipation can lead to impaction with overflow incontinence or simply constant elimination of hard stool with physical movement. Treating the constipation aggressively is the first step in continence management. An effective bowel maintenance program needs to be completed at regular, consistent times as part of the person’s routine. Forgetting to perform any portion of a bowel program on time or at regular intervals will make it ineffective, and incontinence may likely occur. The app reminds patients to use their medications, enemas or other interventions important for maintaining bowel health. The app’s display allows the patients to input what type of problems they are currently having with their bowel movements, such as diarrhea, abnormal color, blood, pain, no production of stool, or the option to enter free text for another problem.

### Clinician Portal

The clinician portal is a Web-based portal system designed for clinicians to engage and monitor patients. In this study, the clinician portal served 3 primary purposes: monitoring patients’ conditions and adherence to regimens, prescribing regimens or treatment plans, and communicating with patients. The primary users of the health portal were the WC’s directly supporting patients (typically social workers, nurse coordinators, case managers, and/or patient advocates). The secondary users of the clinician portal were physicians, who were consulted by the WC’s when interventions required their direction or clinical supervision. Clinicians used desktops, laptops, or tablet computers to access the health portal. Using the health portal, the WC could engage and monitor patients from her desk at the clinic, at the office, or at home. A typical work day of the WC would start with accessing the dashboard in the health portal to get the latest status of the patients under her care. The dashboard would provide a graphical overview of all patients and provide highlights of the small group of patients that require attention (low adherence to regimens, new wound, refill of medications needed, etc), prioritizing tasks. The WC would subsequently focus on this group of patients by looking at the detailed monitoring data of the individual patients. After evaluating the individual monitoring data, the WC could engage patients by prompting the patient via apps, secure messaging, or phone. The WC and physician can regularly discuss patient cases and update the regimens, self-care schedules, and medications. The update of self-management tasks or medications can be conducted using the health portal and then pushed to the smartphone apps in real time.

### Bi-Directional Data Exchange Between the Apps and the Clinician Portal

The key to a successful self-management program is the clinician’s ability to engage patients by monitoring progress and sending regimens to the patients’ apps. A two-way data exchange protocol was needed to support this health care and self-care model and address major weaknesses of the existing apps and mHealth for chronic conditions [30]. The focus of interactions between patients and clinicians in the iMHere system was to promote self-care and adherence to regimens. The novel protocol needed to have apps with the capability to work independently when a data connection was not available. This feature was necessary for those in rural or low-resource areas who had a spotty data connection and necessary for users with no data plan. The protocol also needed to support an innovative security mechanism which would erase data if the phone were lost, because security and confidentiality are of paramount importance in a mobile health application. The smartphone apps were designed to work as independent local apps, yet they were able to synchronize with the health portal in real time when an Internet connection was available. The protocol enabled a seamless exchange of monitoring data from apps to portal and of treatment regimens from portal to apps.

### Usability Study

The usability study was conducted in 3 phases. The objective of the usability study was to find usability problems and refine the design of the system to address the problems found. Previous studies from the human-computer interface (HCI) literature found that 80% of usability problems can be found with only 5 subjects [35-38], with almost all of high-severity usability problems uncovered with only 3 subjects [35]. The estimated required sample size for a usability test depends on the problem space [39]. Since the self-care tasks in iMHere apps are well-defined, the problem space is not as large as that of common software systems. Due to the existence of well-defined tasks [39], the involvement of more than 1 evaluator (all co-authors) in the studies [40], and the fact that the study had 3 phases [35,37], the sample size of 14 participants (8 in Phase 1, and 3 in Phases 2 and 3 respectively) can be considered sufficient for discovering usability problems in the system.

The first phase was conducted in a natural environment by giving smartphones to patients for a few weeks and asking patients to use the apps, which were not yet connected to the portal. The purpose of the first phase of the study was to evaluate the usability of the apps in supporting self-care, focusing on the issues related to self-care workflow. The second phase was conducted in a controlled (lab-like) environment where patients were asked to perform specific tasks while their performance was observed and measured. The third phase was conducted in a natural environment like phase 1 but focused instead on the communication between the apps and the portal.

Phase 1 involved 8 participants, while phases 2 and 3 involved 3 participants. All participants in the usability study were individuals with SB, who are potential users of the iMHere system. More specifically, participants were 18-40 years of age with SB and hydrocephalus but no diagnosis of intellectual impairment or serious mental illness. None of the subjects reported or demonstrated sensory (eg, vision, hearing) or motor deficits, which would interfere with operation of the smartphone device. All subjects were cell phone users prior to participating in the study. These subjects had already participated in the in-person SBAWP Wellness Program. Therefore, these participants had experience participating in a preventative wellness program and performing self-care tasks to reduce secondary conditions. This prior knowledge enabled them to better understand the concept of using proactive measures to avoid and manage health problems.

## Results

### Smartphone Apps

**Figure 2:**
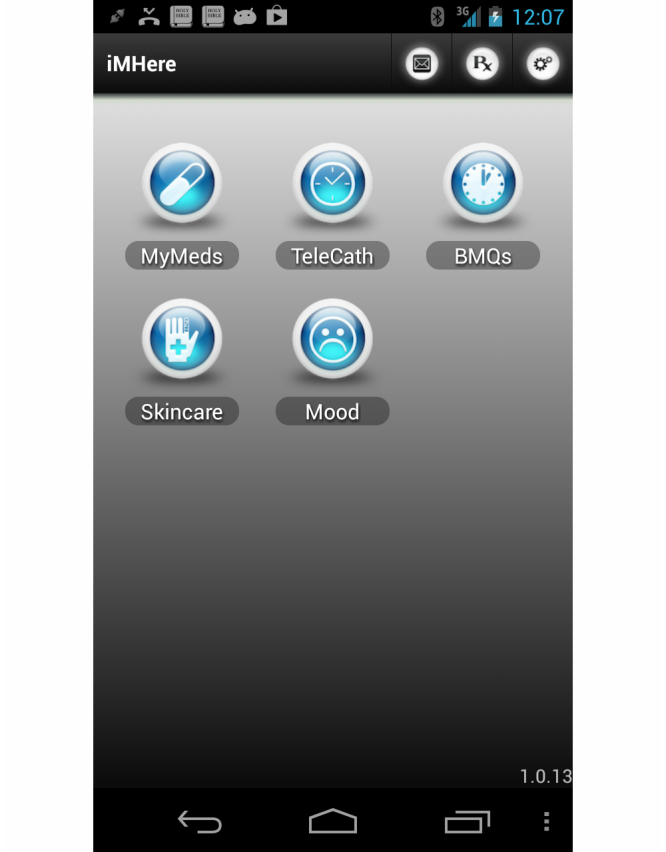
Home screen for suite of apps. App selection area launches individual apps and common action bar initiates tasks within all apps.

The first result of the study is a complete iMHere system that is ready for clinical trial. The iMHere on the smartphone is a suite of apps, currently consisting of 5 apps, as illustrated in Figure 2. Every app included in iMHere allowed patients to schedule reminders, sound alarms, and collect information when reminders are scheduled. Scheduling and responding to these reminders work the same way across all of the apps, simplifying operation. Similarly, most other tasks within iMHere started through a common set of Action Buttons with consistent behavior across the 5 iMHere apps.

The home screen of the iMHere suite consists of 2 main areas: the action bar and the app selection area. The action bar is located at the top of the home screen, as well as all app screens, and contains action buttons that access most of the functions of iMHere. The area to the left of the action bar contains current location (main screen, apps, etc), and the area to the right of the action bar contains the action buttons that control the tasks. The individual apps from within iMHere are launched from the home screen by clicking their respective icons in the app selection area. Clicking on an icon in the app selection area takes the user to the respective app’s main screen. Each app in turn behaves in a familiar way through the common set of Action Buttons located on the Action bar. The following is a description of the apps in the apps suite.

### MyMeds: An App for Medication Management

**Figure 3:**
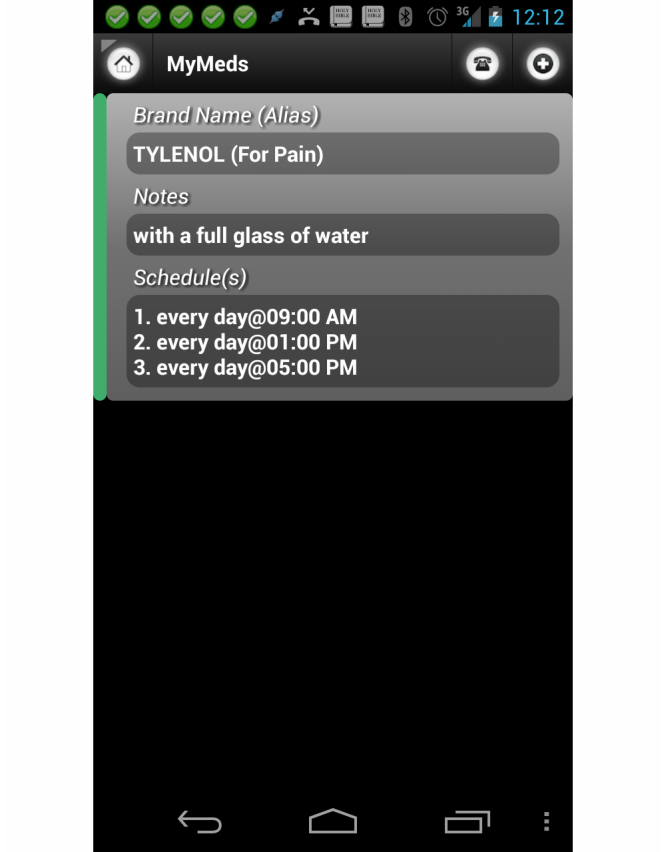
MyMed app with 650 mg Tylenol scheduled every 4 hours.

MyMeds keeps track of medications and their schedules, and provides reminders of the medication schedules. The medications and their schedules can be entered by patients from the apps or can be sent by the clinician from the portal. The app allows for complex, multiple-drug schedules, and also allows users to keep drugs on the list that they are no longer taking. This unscheduled drug feature is useful when a patient stops taking a certain medication for a period of time, but expects to return to using it. The reminder shows the name of the drug, the dosage, its purpose (such as “for pain”), how many tablets to take, how frequently to take the medication, and any notes that might help the patient identify the specific drug. Please refer to Figure 3.

Entering drug names can be fraught with mistakes. To help avoid errors in drug name and dosage, we use the drug database from National Drug Code (NDC) of the US Food and Drug Administration (FDA) [41] and provide users with autocompletion and auto correction when a user enters drug data. The result is that the drug and its dosage are from the known entity in the NDC database, unless prescribed differently by the clinician from the portal.

### Skincare App: An App for Prevention and Caring for Wounds

**Figure 4:**
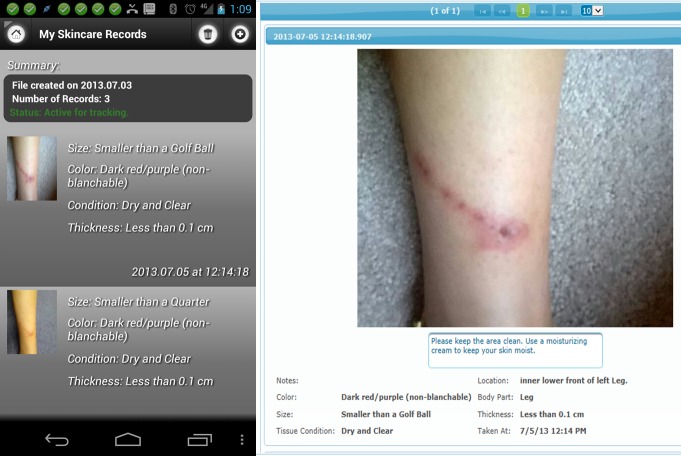
Skincare app and portal side by side.

The Skincare app provides reminders to perform the task of visual inspection of one’s skin as a preventative measure, often termed a “skin check.” Persons with insensate areas of the body should perform skin checks at least twice per day. The Skincare app also allows patients to track the progress of skin problems when detected (such as pressure ulcers or lacerations) by taking pictures with the smartphone’s digital camera. Pictures can be taken periodically for comparison, and photos are stored on the phone for the patients’ reference, as well as synched through the secure portal for clinician review (illustrated in Figure 4). Skincare can track as many skin problems as needed, keeping photos from each separate skin problem organized together as a single case. Daily reminders can be set to perform skin care checks and to take additional photographs for comparison.

**Figure 5:**
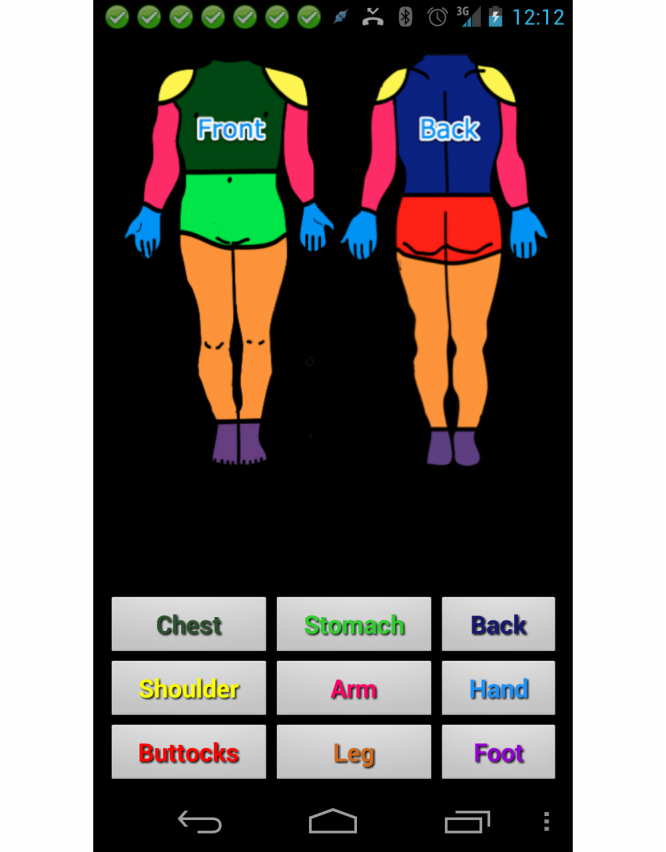
Skincare app affected area selection.

A human body graphic provides the user a way to indicate which body part is affected by a wound (illustrated in Figure 5). After the user clicks the body part, the app lets the user upload a picture of his or her skin condition and update the problem as needed. After the user takes a picture of his or her skin condition, there are questions about the condition that need to be answered so that the clinician can have more detailed information about the problem. This detailed information is also used to flag photos that warrant immediate attention from clinicians. Some of the questions include: color of the wound, size of the wound, amount of drainage, or visual details about how the wound looks.

The app also provides a tool to help the clinician and patient track changes in skin care problems. The skincare app organizes the pictures into cases and records them to facilitate the tracking of multiple problems. Each skincare problem is a case. Within each case there may be many records or pictures. The organization of pictures into cases allows the clinician and patient to easily track progress by comparing the pictures in each case. Related pictures from a problem are organized together, in a sequence over time, so that changes can be noted by the clinician and patient. The Skincare app main screen shows all cases, with a picture of the most current record from each case. The patient can click a case and the app will show all of the records for that skincare case, starting with the most recent.

### Mood: An App to Track Mental Health Status

**Figure 6:**
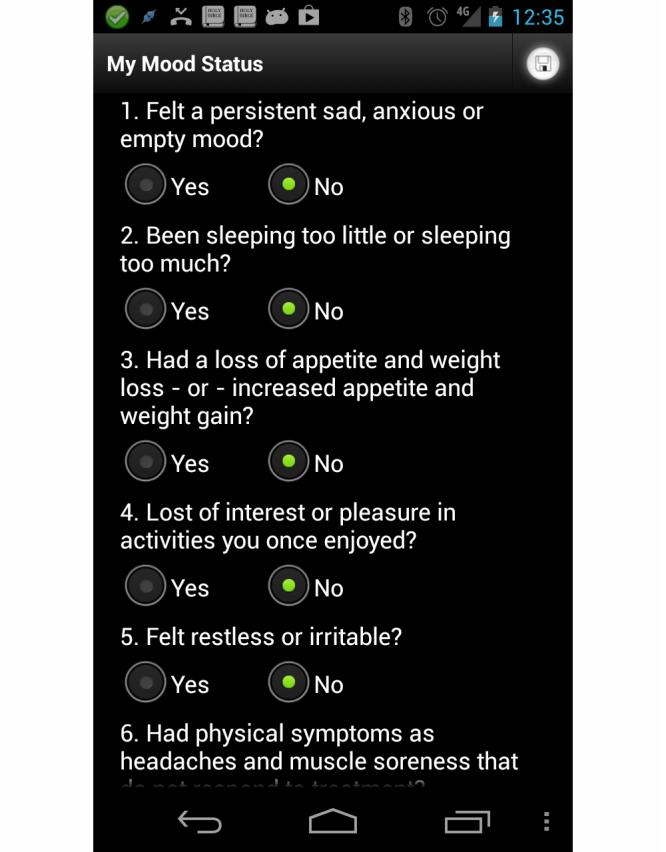
Mental health app mood survey.

This app lets a patient or clinician schedule routine mood questionnaires or take them on demand. The questionnaire is based on the depression screening developed by Mental Health America, formerly known as the National Mental Health Association [42]. This 10 items survey asks questions to determine the patient’s mood, including whether the patient has: been sleeping too little or too much; had thoughts of death or suicide; had a hard time concentrating, remembering, or making decisions; had a loss of appetite and weight loss; or increased appetite and weight gain; et cetera. The app records the results of the questionnaire and sends them to the clinician. The Mood app gives an immediate warning to the patient, and provides quick access to a patient’s local mental health crisis line if the score on the questionnaire gives cause for immediate concern. Please refer to Figure 6.

### TeleCath: An App for Bladder Self-Catheterization Management

TeleCath allows a patient or clinician to set daily reminders for bladder catheterization and to schedule daily inquiries about urinary incontinence. Any problems with catheterization (such as pain, difference in urine color, cloudiness, blood in the urine, or lack of urine output) can be reported along with the patient’s response to the reminder to catheterize. All information regarding catheterization problems or incontinence are synchronized through the clinician portal and made available for review by clinicians.

### BMQ: An App for Bowel Management

BMQ allows a patient or clinician to set daily reminders for performing bowel management (defecation) and to schedule daily inquiries about defecation or incontinence. Any problems with bowel management (such as diarrhea, blood, pain, etc) can be reported along with the reminder. All information regarding defecation problems or incontinence are synched through the clinician portal and made available for review by clinicians.

### Clinician Portal

**Figure 7:**
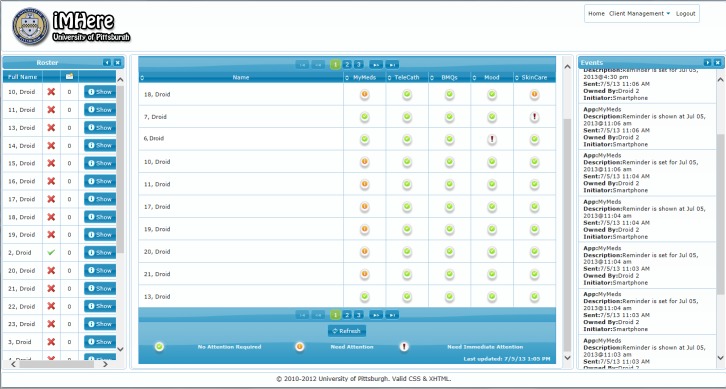
Clinician portal: roster table displays a list of registered patients and their connection status. Dashboard displays priority flags for each patient and application.

We developed the clinician portal to allow clinicians to send information to patients’ apps, including personalized self-care plans, medication data, and phone numbers of pharmacies to call for refills. The portal is illustrated in Figure 7. The main components of the portal are the patient roster, dashboard, and utility bar. The roster was designed to mirror the smartphone apps. It allows the clinician to create self-care plans for the patients (prescribe medication, schedule skincare, and TeleCath, etc), and review data sent from the apps. The dashboard was designed to provide the clinician with a decision support system and with analytics that can enable the clinician to triage the needs of a large caseload of patients. The utility bar is used for tasks not directly related to patient care, such as client (patient smartphone) management and authentication management.

The Patient Roster is a list of all the patients registered for the clinician. Role-based access is used to give clinicians access to only those patients for whom the clinician is providing care. The patient roster provides information about the patients’ smartphone connectivity, with a green check indicating that a patient’s phone is currently connected, and a cross indicating that it is not connected. The roster provides clinician access to the detailed patient data. The dashboard is a grid that allows an “at a glance” review of clients’ activity and allows the clinician to quickly navigate to areas that need attention. All registered clients are shown on the dashboard, and their activity for each app is classified as: no attention required, needs attention, needs immediate attention. The clinician can review the corresponding patient by clicking an icon on the grid.

### Two-Way Communication Protocol

The iMHere two-way communication protocol was developed by extending the Extensible Messaging and Presence Protocol (XMPP) standard [43]. The XMPP protocol was designed with the assumption that the Internet connection would be constantly stable and always available. A mechanism in the application layer to deal with unstable connections in the wireless network was developed in the iMHere protocol to make it usable by patients living in areas where the connection is not always stable. The first major extension is the capability to support efficient and reliable communication that can work under various connection qualities, including when connection is poor or completely unavailable. The communication protocol allows the portal to detect if the mobile device is active and report it to the patient roster.

The second extension is the implementation of advanced security features. Security and confidentiality in a mobile health application is of paramount importance. The authentication process requires a combination of the device’s phone number and the device’s unique equipment number. All the traffic between the smartphone and the portal is encrypted. The Advanced Encryption Standard (AES) established by the United States National Institute of Standards and Technology (NIST) [44] 128-bit is used for all data communications between the smartphone and the clinician portal. No identification is attached to the data in the smartphone. The communication protocol also allows the clinician to lock the apps and to delete all data in the smartphone in the event that a smartphone is lost. The data will still be preserved in the portal and can then be reconfigured to the apps. HIPAA has not specifically designated guidelines or compliant regulations for telehealth technology. In accordance with existing HIPAA regulations for other technological use and exchange of health information via Internet technology, iMHere is considered HIPAA compliant due to its security measures, privacy precautions, and use of a covered entity to store encrypted data.

### Usability Study

The initial design and implementation of iMHere at the first stage was based on clinicians’ predictions and developers’ understanding of what patients need to encourage self-care. Extensive testing had been conducted with a research group to simulate real use by patients. However, the predicted scenarios from a research perspective may not have been adequate to cover all activities in real use. In order to address any unknown issues with the meaningful use of iMHere, a group of target users (patients with SB) were introduced to usability study phase 1. The first 2 phases of the usability studies uncovered a few usability issues related to the self-care tasks and navigation.

#### Phase 1: Focus on Self-Care Workflow

The first problem was related to the scheduling, which was originally designed to be object-centered (such as medication) instead of patient-centered. In the medication app, a separate alarm would ring for every medication because each medication is entered and scheduled separately. It was not convenient to have multiple alarms ringing at the same time. We discovered that a better design is patient-centered: all medications included in a person’s regimen should be scheduled using a reminder for all medications that need to be taken at a specific time. For example, a patient who needs to take 3 medications 3 times a day can set up reminders for Morning (8:00 AM), Afternoon (1:00 PM), and Evening (7:00 PM), with each reminder applied for all 3 medications. The same concept should also be applied to the response to a reminder. When the patient accesses the app after receiving the reminder, the patient should see a list of medications to take. The patient could respond by pressing a check box next to each medication to indicate that the dose was taken. If the patient does not or cannot take the medication, a place to indicate the reason why can be included and this information could then be immediately reported back to the WC through the portal. In relation to the scheduling and reminders, the snooze function and repeated alerts have been removed. Instead, if the patient does not respond to a reminder, it will be put into a missed schedule list that the patient can access. The missed schedule list will appear as a notification, similar to a “push” notification in email or digital calendar.

The second problem was related to the frequency of self-care tasks. This problem was uncovered as a result of more intense discussions with the clinicians, not from the patient studies. The apps for BMQ, Mood, and medication were originally designed with daily schedules in mind, but some of the self-care tasks are not performed on a daily basis. Patients who complete bowel program regimens typically perform this self-care task every other day or every third day. The mood questionnaire is typically needed only 1 time per week. There was also a need for the ability to take medication less frequently than on a daily basis; on certain days of the week, once per month, et cetera. A patient may also need to vary the time of day to perform bowel management program if it interferes with other activities or is reliant on a caregiver to assist them with performing this task in some way. Appropriate changes in alerts were made to accommodate these needs.

#### Phase 2: Focus on Navigation and User Interface

We also found issues related to navigation and the user interface during the first phase of the usability study. We conducted a second phase of the usability study to focus on these issues. The term “user-interface” in this study is used to refer to the presentation of elements that are directly accessed by users; such as button size, text size, and colors. The usability study was conducted in a controlled environment where patients were asked to perform tasks and detailed problems were observed.

Due to the narrow width of the scrollbar, it was not obvious to the patients that they needed to scroll down, leading to under-completion of the data. Related to this problem was the fact that the green “+” button, used throughout the app suite to add items such as new schedules, also required patients to scroll down the screen to use it. Another issue was found with the Skincare app, where an on-screen button was used for taking wound pictures. This button was difficult to use for patients attempting to photograph a wound on a less accessible area of their body. Instead of using an on-screen button to take the picture, patients could more easily utilize the physical control button on the device that is already built in to perform this function. We also found minor issues related to the use of a light text color on top of a grey background making text difficult to read; also an activity button at the top of the screen was too small for some users to access.

In addition to addressing these issues by redesigning the apps, we also identified the need for more user training for some of the apps. MyMeds and Skincare are relatively complicated apps that accounted for 90% of the errors (25 out of the 28) that the users made during usability study phase 2.

#### Phase 3: Focus on Patient-Clinician Communications

Phase 3 of the usability study was conducted to focus on the two-way communications between the apps and the clinician portal. The goal of this implementation was to evaluate problems in the clinical service delivery model and to address the problems before moving on to a full-scale clinical implementation. We encountered problems with the implementation of XMPP protocol in a 4G wireless connection. The 4G wireless signal is not always stable, and the signal can be lost or work only intermittently in some areas. The iMHere protocol was designed to handle an unavailable connection. However, a problem that we encountered was the inability of the XMPP protocol to accurately detect the availability of a connection. This situation led to packet loss when the app would attempt to send data despite the absence of a connection. We added 2 mechanisms to improve on the reliability of the XMPP protocol:

1. *App to portal transmission.* The first mechanism was to verify the connection’s availability before sending data. This mechanism was to ensure that the data would be sent only when the connection was definitely available or otherwise be stored locally for later transmission.
2. *Determining wireless signal strength*. The second mechanism was developed to inform the portal about the device’s actual signal condition (portal to app transmission). This mechanism ensures that the portal would send data to the device only when the device was receiving a good wireless signal. When the device was receiving poor signal strength, it would notify the portal to hold the data until the signal strength improved.

Extensive testing in various signal conditions was conducted by having smartphone apps send data to the portal and by having clinicians send treatment plans to the patients. The result is a reliable two-way protocol that works under any signal condition and in any version of the Android operating system.

### Clinical Implementation

**Figure 8:**
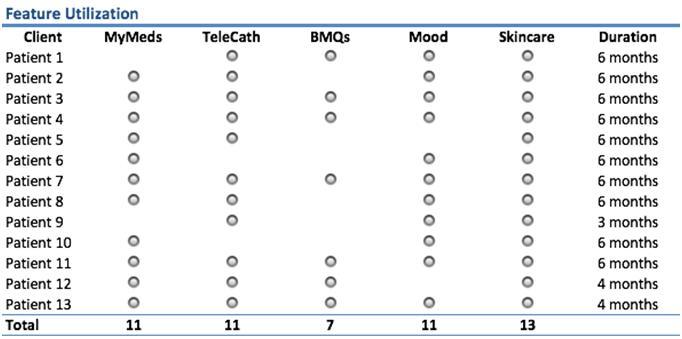
Apps features used by patients.

We implemented the system on a rolling basis. In the first 6 months of the clinical implementation, 14 patients used the system, with the length of participation varying between 3 months (1 patient) to 6 months (10 patients). Figure 8 shows the duration of usage for each patient. The patients were located in a tri-state area (Pennsylvania, Ohio, and West Virginia) around Pittsburgh. More than half of the patients were located in rural areas with the furthest distance being a 2.5 hour drive from Pittsburgh. There was 1 patient who dropped out because of a very poor cellular phone signal in the area where the patient lived (a basement apartment in a rural area). There was no drop out for reasons other than a poor signal.

The smartphones and the data plan were provided by the study. The Android operating systems used include versions 2.3 to the latest, 4.1. During the implementation, we did not find serious problems with the fragmentation of the Android operating system other than glitches with the picture-taking function of the Skincare app. We found that picture-taking in the Skincare app did not work in the Android 4.x, despite working well in Android 2.x. After debugging, we found that Android 4.x has faster threads in the operating system, which are not synchronized with the components related to the smartphone camera. The result is that the picture object could not be captured by the apps. We made changes in the Skincare app’s code to solve this issue. In order to deal with the operating system fragmentation, the iMHere system is designed to use only the core libraries from Android 2.3 (the first Android version that was widely used). The libraries run on any later Android versions without any problem. Implementing bi-directional communication between smartphone apps and a portal in a clinical setting is a complex process, more complex than implementing a smartphone app or a clinician portal on its own. This is because any change in the apps has to be coordinated with the related change in the portal, and this requires the involvement of both patients and clinicians. With respect to connection type, we found that the 4G wireless connection is still not as reliable as a WiFi connection, but we anticipate that the situation will improve with time. Overall, we developed a robust protocol that is efficient and capable of handling unreliable connections.

### Usage of the iMHere System

A patient may use any number of app features from those available in iMHere. Figure 8 provides a matrix showing patients and the apps features used by the patients. There were 5 patients who used all the app features, 3 patients used all except 1 of the app features, while 4 patients used only 3 app features. Patients were trained on how to use the apps, how to set up regimens, and how to manage self-care regimens on their own. Patients knew how to revise their regimens if something changed in their personal plans or if they received recommendations from a physician outside the spina bifida clinic. For instance, a patient may choose to modify the app for their bowel program so their schedule may fit better with a family gathering for that particular week. Figure 8 illustrates the features used by patients at the start of the implementation. Since patients have all the app features on their phones and were trained to use all the features, patients could decide to change the usage along the way. Patients were able to add features or drop features at any time, although the frequency of change was low in the first 6 months. For example, Patient #8 started to use the BMQ app feature on the fourth month of the implementation.

**Figure 9:**
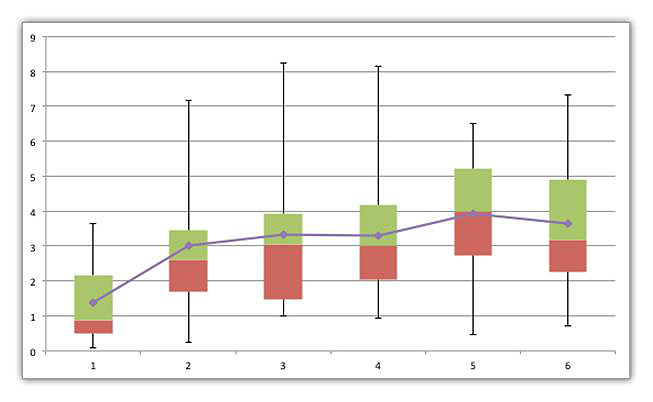
Average daily usage per day per patient.

The average daily usage per patient of the iMHere system is illustrated in Figure 9. This figure includes the usage of all app features (from MyMed to Skincare). We observed that the daily usage increased significantly in the first 2 months (from approximately 1.3 times a day to over 3 times a day). This is consistent with how patients began using the apps. Not all apps were in full use until the second month because the patients were trained one app at a time until it was being utilized properly in the first month. The usage did plateau after 2 months, at around 3.5 times a day per patient. This pattern of increasing usage in the first 2 months and the subsequent plateau is relatively consistent in all patients. The pattern holds when data from all patients are included, as well as when the 2 outliers are excluded. The data shown in Figure 9 are data with the 2 outliers (patient with the highest usage and patient with lowest usage) excluded. Patient #10 had the highest usage, with an average usage of 13 times per day, while Patient #6 had the lowest usage with an average usage of once every 3 days. Patient #10 had the highest usage because of the number of medications that needed to be tracked (15 medications), while Patient #6 had the lowest usage because the patient had social issues not related to the clinical intervention. As mentioned above, after the second month, the usage of every patient was relatively consistent.

### Utilization by App Feature

**Figure 10:**
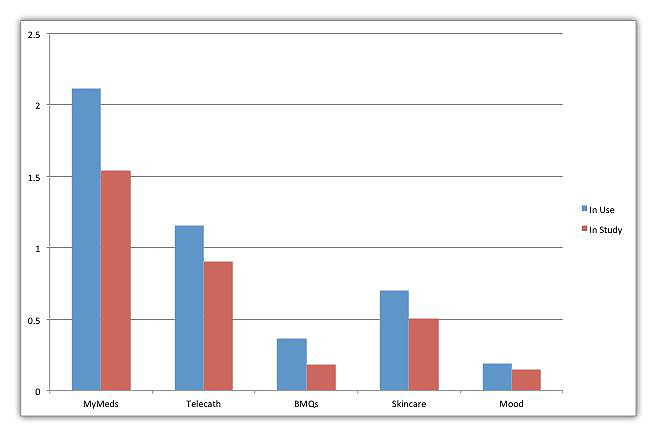
Average daily usage of app features.

The breakdown of utilization per day is illustrated in Figure 10. The first bar in the bar chart is a utilization per day per patient for those who used it (patients who were not provided with the app were not included in the calculation), while the second bar is the average utilization of the app feature for all patients in the study (including those who did not use it). It is important to note that patients were given only those apps which were relevant to their medical problems (eg, if patient did not need a bowel program, he was not given BMQ app). The second bar is shown to make the numbers consistent with the average daily usage per patient in Figure 9.

The MyMed app was the most frequently used app, with a usage of more than twice a day. This feature was used by all but 2 patients to manage their medications. Of the 11 patients who took medication, 6 used only 1 medication, 5 used 2 medications, and 1 patient each used 5 and 15 medications. The app feature used least frequently was the Mood app, at about once every 4 days. This is to be expected because the Mood feature that measures depression is usually administered once a week. The second most frequently used was the Telecath app feature, used about 1.7 times per day. This app feature includes a once-per-day questionnaire asking whether the patient is having any problem with incontinence and, if so, how many times it happens in 1 day. The BMQ was used less frequently than TeleCath because the bowel program is done less frequently than bladder self-catheterization. It is interesting that the Skincare app feature was used quite frequently. Most patients used the Skincare app feature as a reminder to conduct skin self-care tasks because they did not have serious skin conditions. However, there were 4 patients who had serious skin conditions that necessitated their taking pictures of the wound and sending them to the clinicians. The clinical phase of this study is still on-going and additional clinical outcomes will be reported at the conclusion.

## Discussion

All patients from the usability and clinical intervention studies were interested in using iMHere and making the apps part of their daily routine. Other than the minor usability issues, they were satisfied with the user interface and felt comfortable using the application. The patients stated that it required only a small amount of training time before a user is able to use the system. This result suggests that the goal of developing iMHere as a self-care tool has been accomplished. This acceptance also provides a good base for allowing clinicians to monitor patients’ conditions and intervene sooner to prevent secondary complications. The results of the implementation indicate that the iMHere system has been used as expected. Patients used the system consistently and actively during the first 6 months of the clinical implementation. The full study is a year-long in duration.

The innovative iMHere system can potentially reduce the burden of care for providers, reduce the cost of supporting the wellness service, and empower patients to conduct effective, timely, preventive self-care. The iMHere system allows clinicians to send self-care regimens and adjust existing treatment regimens without having patients come to the clinics. These innovations may help reduce the cost of care for people with chronic conditions and improve health outcomes by reducing secondary complications such as wounds or infections. The iMHere system was designed using concepts found to be extremely effective in managing patients with complex chronic conditions within a medical home model [9]. The iMHere platform was designed to be scalable to allow support service delivery for patients with various types of chronic conditions, but it can also be implemented in clinics for patients with other chronic conditions and cognitive deficits, such as cerebral palsy, multiple sclerosis, traumatic brain injury (TBI), or those with medically complex conditions such as diabetes or HIV/AIDS.

The data in the iMHere portal can potentially be integrated with the electronic health record (EHR) system of medical centers or clinics. This integration would allow health information pertinent to self-care, such as lab results, to flow to smartphone apps, and allow a summary of patient conditions to flow to the EHR system. In the future, health records will not only contain data generated by providers (doctors office, lab, hospitals), but also data generated by patients. The iMHere system collects this new patient generated data. This is a challenge for future EHR systems since the amount of data and frequency of data will be much larger than that of the existing EHR. We envision that only a summary of the patient generated data will be integrated into the EHR system, since clinicians who are not directly involved in managing patients with chronic conditions will not be using detailed monitoring data from patients. The challenges in managing large amounts of high-frequency data generated, by patients (including monitoring data) and in summarizing the large data for integration with the EHR system are potential areas of research opportunity for big data analytics.

## Abbreviations

AES: advanced encryption standard
BMQ: Bowel Management App
CIC: clean intermittent catheterization
EHR: electronic health record
FDA: US Food and Drug Administration
HCI: human-computer interface
HIPAA: Health Insurance Portability and Accountability Act
mHealth: mobile health
NDC: National Drug Code
NIDRR: National Institute on Disability and Rehabilitation Research
NIH: National Institutes of Health
NIST: US National Institute of Standards and Technology
SB: spina bifida
SBAWP: Spina Bifida Association of Western Pennsylvania
TBI: traumatic brain injury
UTIs: urinary tract infections
WC: wellness coordinators
XMPP: Extensible Messaging and Presence Protocol
